# What Do Parents Think about Chromosomal Microarray Testing? A Qualitative Report from Parents of Children with Autism Spectrum Disorders

**DOI:** 10.1155/2016/6852539

**Published:** 2016-06-20

**Authors:** Lei Xu, Linda Crane Mitchell, Alice R. Richman, Kaitlyn Clawson

**Affiliations:** ^1^Department of Health Education and Promotion, College of Health and Human Performance, East Carolina University, Greenville, NC 27858, USA; ^2^Department of Child Development and Family Relations, College of Health and Human Performance, East Carolina University, Greenville, NC 27858, USA

## Abstract

*Background*. Chromosomal Microarray Analysis (CMA) is increasingly utilized to detect copy number variants among children and families affected with autism spectrum disorders (ASD). However, CMA is controversial due to possible ambiguous test findings, uncertain clinical implications, and other social and legal issues related to the test.* Methods*. Participants were parents of children with ASD residing in the North Eastern region of North Carolina, USA. We conducted individual, face-to-face interviews with 45 parents and inquired about their perceptions of CMA.* Results*. Three major themes dominated parents' perceptions of CMA. None of the parents had ever heard of the test before and the majority of the parents postulated positive attitudes toward the test. Parents' motivations in undergoing the test were attributed to finding a potential cause of ASD, to being better prepared for having another affected child, and to helping with future reproductive decisions. Perceived barriers included the cost of testing, risk/pain of CMA testing, and fear of test results.* Conclusion*. This study contributes to the understanding of psychosocial aspects and cultural influences towards adoption of genetic testing for ASD in clinical practice. Genetic education can aid informed decision-making related to CMA genetic testing among parents of children with ASD.

## 1. Introduction

Autism Spectrum Disorders (ASD) represent a group of highly inheritable disorders [1]. Although the practices, implementation, and availability of genetic testing for ASD vary in different countries, a growing trend is the embracing of more advanced genetic testing in clinical settings [2–6]. Based on the current clinical guidelines by the American College of Medical Genetics and Genomics (ACMG), an established technology known as Chromosomal Microarray Analysis (CMA) has been recommended as the first-tier test for all children with ASD [7]. CMA is also considered as part of routine care in the ASD diagnostic process and has been available in multiple clinical settings in the United States (USA) such as Duke University Health Systems (DUHS) Clinical Laboratories, Medical Genetics Laboratories at Baylor School of Medicine, and the Division of Genetics and Genomics at Boston Children's Hospital. Furthermore, next-generation prenatal screening for ASD is already under development, although still considerably debated [8].

Research on the clinical benefits/usage of CMA has shown that this test can potentially help ASD patients and their families to explain the causal link of genetics and autism, predict the recurrence risk, help develop timely treatment (medical, educational, and behavioral) plans, and guide parental decision on reproductive options [9–13]. Moreover, compared with traditional tests, CMA has the following features: (1) CMA offers the highest detection rate (approximately 12–19%, compared with G-banded Karyotyping at <3%) [14]; (2) CMA has the ability to detect extra or missing segments of genetic material; therefore, it can provide evidence for other neurological conditions associated with ASD [4]; and (3) CMA test results can influence the medical management among the test-taker who underwent CMA and received abnormal test results [15].

Although CMA has proven to be a more powerful technology in detecting genetic variations over conventional cytogenetic tests [14], this emerging test might raise potential ethical and social considerations, such as genetic discrimination, insurance concerns, confidentiality issues, ambiguous explanation of the test results, and psychological burdens related to the test [12, 13, 15–18]. For instance, in a recent study conducted by Reiff et al. [13] among parents of children with ASD who had CMA testing, parents expressed concerns about the limited usage of the test, the misunderstandings and ambiguities of the test results, and the negative emotions (e.g., “a lot of guilt”) associated with the test results. It was also noted that clinicians and other providers who order the test might face challenges in sharing the extensive details from the tests with the test-takers. In addition, as Riggs and colleagues pinpointed [18], genetic health care professionals might have reservations to order CMA for their patients due to both practical and perceived barriers. The reasons included the difficulty of interpreting test results and other negative impacts on the test-takers, for instance, lack of “actionable” medical plans after the test and the denied coverage by insurance companies. Although reimbursement status for CMA genetic testing varied in the US, who would pay for the test remains a big concern among patients and families who intend to undergo CMA genetic testing.

Albeit CMA has both potential benefits and harms, parents' awareness and interest in taking their children with ASD to undergo CMA are still largely unknown. To ensure adequate access to genetic technologies and to reduce the potential negative impact of concerns regarding CMA, there is an immediate need to understand perspectives from parents of children with ASD regarding their awareness, motivations, and inhibitors regarding undergoing CMA testing, including testing themselves and their affected children.

This study focused on parents of children with ASD in Eastern North Carolina (ENC), which is rural area that serves a largely diverse, agricultural, and economically challenged area of the state. ASD identification in North Carolina (1 in 58) is higher than the national average (1 in 68) [19]. This geographical location has one of the highest rates of families who are Medicaid-eligible (Medicaid is a joint federal and state program in the US which helps people with limited income and resources), disadvantaged parents who have children diagnosed with ASD.

This study was part of a larger pilot research project investigating the genetic literacy, education needs, and decisions with regard to CMA genetic testing among parents of children with ASD in ENC. We sought to address three main questions in this particular study. (1) Are parents of children with ASD aware of the availability of CMA testing? (2) What are motivating factors for parents to participate in CMA testing? (3) What do parents identify as barriers that would keep them from participating in CMA testing? The questions we asked containing parents' motivations and barriers associated with both taking their children to undergo the test and test themselves. Our investigations specifically related to parents' perceptions on undergoing prenatal CMA were not reported in this paper. Parents were informed that their answers should be provided in a hypothetical scenario.

## 2. Materials and Methods

Based on the current literature and our previous published work [13, 20–23], we developed a multipart, semistructured interview guide with 14 questions examining parents' perceptions regarding CMA genetic testing. The questions covered general information regarding CMA genetic testing, inquiries about parental knowledge, perceptions, family planning, and communication related to CMA genetic testing. We used individual, face-to-to face interviews to elicit information about parents' perceptions and decision-making about CMA genetic testing. This study was conducted between December 2014 and February 2015.

The reasons we adopted this qualitative research method are threefold: (1) qualitative methods are more relevant for research areas that have not been adequately researched before, (2) qualitative methods are advantageous in procuring in-depth information about a largely unknown topic, and (3) qualitative methods are powerful in understanding a complex decision-making process [24] such as the one examined in this research.

### 2.1. Sample and Recruitment

A snowball sampling technique was used to recruit parents of children with ASD living in ENC [24]. In addition, we also reached our potential participants through verbal advertisement at a local autism research fair for parents of children with ASD and through flyers sent to parents through the Autism Society of North Carolina and other local autism education agencies. Our selection criteria included all parents of children with at least one child diagnosed with ASD residing in ENC. Interested parents contacted the researchers directly and scheduled an appointment for a face-to-face interview. Interviews were also scheduled for couples who were interested in study participation.

Our initial recruitment team was composed of three researchers who specialize in public health genomics, social behavioral sciences, and special education. We also used a train-the-trainer model and recruited one community member who was bilingual (English/Spanish) and worked with Hispanic families who have children with ASD due to the vast Hispanic population that is present in ENC. Prior to implementation, approval was acquired from the Institutional Review Board at East Carolina University.

### 2.2. Procedure

In order to protect confidentiality, participant information was coded using a numerical system. A master list of participants was kept in the office of the lead researcher (LX) in order to complete reality checks during data analysis.

Prior to the interview, the purpose of the study as well as the consent document for participation was reviewed with each participant. We also provided brief information regarding CMA genetic testing at the beginning of each interview session. Interviews lasted 45–60 minutes. Information was provided in Spanish for those who were non-English speaking. The community bilingual specialist conducted the interviews for Spanish-speaking participants. All interviews were audio recorded in order to provide a reliability check for information. Researchers also collected field notes during the interviews to ensure that all related information was collected. Each parent received $40 gift card for participation.

### 2.3. Data Analysis

Each interview was transcribed verbatim and analyzed using Nvivo 10 [25]. We used a thematic analysis approach, an inductive method, to analyze our qualitative data. The feature of this analytical approach was exploratory and theory-driven, rather than depending on preexisting coding themes. The research team members met weekly to define and redefine the thematic categories as well as resolve discrepancies when divergent opinions arose in the course of data analysis. This researchers' triangulation strengthened the reliability of our data [24].

## 3. Results and Discussion

### 3.1. Sample Characteristics

As illustrated by Table 1, the majority of the 45 participants were mothers (60%, *n* = 27). Parents had a mean age of 39.1 years. More than half of participants were spouses who were interviewed individually (57.8%, *n* = 26). Almost 56% of participants were White and the remaining were minorities. Slightly over half of the parents (57.8%, *n* = 26) had an annual income lower than $55K. Forty-four children (35 boys and 9 girls) with ASD were involved in this study and they represented diverse severity levels of ASD as reported by parents (Table 1). Guidelines provided by DSM-5 by American Psychiatry Association were used to assign severity levels of ASD with Level 1 representing mild characteristics of autism, Level 2 moderate, and Level 3 severe [26]. Among the 44 children involved in this study, 9% (*n* = 4) were in Level 1 (high functioning in social communication and less severe symptoms of repetitive behaviors), slightly more than half (52%) were in Level 2 (high functioning in social communication and moderate symptoms of repetitive behaviors), and a quarter of the sample (25%) were in Level 3 in the severe category (either with very low function in social interactions or with the need for very substantial support). Six parents were unable to define the severity level of their children's ASD which accounts for 14% of the children. When parents provided the information regarding severity level of ASD, three of them also mentioned that their children also had intellectual disability. According to the parents, none of them knew any identified etiology of their children's autistic conditions.

### 3.2. Interview Findings

Based on parents' perspectives on undergoing ASD genetic testing, three main themes emerged from the data: parental awareness of CMA genetic testing, test motivations, and perceived barriers to undergoing CMA genetic testing.

#### 3.2.1. Parental Awareness of CMA Genetic Testing

Before we asked parents their attitudes toward CMA genetic testing, we asked parents “what is the first word that comes to your mind when you hear the word genetics.” Then we provided brief information about CMA (e.g., what did CMA stand for, what was CMA, and what is the testing procedure). Regardless of the diverse socioeconomic status, none of the 45 parents reported that they were knowledgeable of CMA. One of the fathers with twin boys who was highly educated and had previous training in molecular genetics reported that he never knew that CMA was a genetic test that was available to his children (twins) with ASD. According to him,

*This particular test I have not heard of. I've heard of general genetic testing, but not CMA.*

Another mother who also doubted that anyone in the local autism community had heard of the test before commented that

*No, not in the group here, local – I don't know anybody that has (heard of CMA genetic testing). *

#### 3.2.2. Test Motivations

Table 2 illustrates recurrent motivations for why parents reported that they would participate in CMA (including testing themselves and their children with ASD). The subthemes, percentages, and illustrative quotes are provided in Table 2.

*Helping Identify a Potential Cause of Autism*. The most recurrent motivation from participants was whether CMA genetic testing could help identify a potential cause of autism. Almost half of participants (*n* = 21, 46.7%) mentioned that the ability for CMA genetic testing to provide a link to the cause of their child's autism would motivate them to test themselves and their children. One participant noted that

*Um… to see if there is a genetic link, to see if it's something that I've passed on versus something that I may have caused during my pregnancy. Because as a parent, when you find out your child has autism, your first reaction is- is it my fault? Did I do this? Is something wrong with me? Is something wrong with my child? So, I think a test like that would bring a little bit of peace to you to know whether or not it was in your bloodline, it was in your genetics, and you passed it down. (Male, White, Income: $15–$35K)*

*Preparing for Having Another Child with ASD*. Similarly, being prepared to have a child with autism was mentioned by the equal numbers of participants (*n* = 21, 46.7%). Participants particularly mentioned that CMA testing results would not change their decisions of giving birth to another child, but they still wanted to use CMA genetic testing as a tool to be more prepared for having another child with ASD. One mother specified that

*I think it would be interesting. I would be open to be tested. I don't think that would sway my decisions of whether I would have another child or not, but it might prepare me for the eventually of possibly dealing with another child with autism. (Female, Hispanic, Income: <$15K)*

*Family Planning*. Less than half of the participants (*n* = 18, 40%) indicated that the CMA genetic testing would provide them with assistance in family planning. According to one participant,

*For me, personally, yes, it [testing] would play a major role in family planning just because of my knowledge of a child with a disability, and the cost, stigma, and the opportunity for the child. (Male, American Indian/Alaskan native, Income: >$95K)*

*Early Intervention*. Nearly 40% (*n* = 17, 37.8%) of participants specified that early intervention for their child with ASD would be a motivation to participate in CMA genetic testing. For instance, one participant stated that

*So that would be preparing the boys (autistic children), thinking about early intervention strategies to help the child learn how to communicate and develop. It would give us some more support when speaking with pediatricians and such about hey this is a real concern. What are the things we want to start working on early… so I think those would be some benefits of it. (Male, White, Income: $35–$55K)*

*Research to Benefit Other Families with Autistic Children*. One-third of participants (*n* = 15, 33.3%) indicated that the main motivation to undergo CMA genetic testing would be for research purposes to benefit other families. According to one interviewee,

*I don't plan on having more family… and if it would help other people through my daughter, to know the motive why these things [autism spectrum disorders] happen, I would do it for that reason. (Male, Hispanic/Latino, Income: $15–35K)*

*Benefit Their Own Child with Autism*. A motivation for some participants was that the results of the CMA genetic testing would benefit their child with autism (*n* = 14, 31.1%). These benefits ranged from types of therapies to medical treatments to help their child with autism. One participant indicated that

*Any type of testing that could help his ability to socialize more in the world. Whatever tests are out there or whatever that can help him… Let's do that [CMA testing]. (Female, African American, Income $35–$55K)*

*Getting to Know the Recurrence Risk*. A number of participants (*n* = 8, 17.7%) also demonstrated that a motivation to participate in the genetic testing was the ability to be informed about the recurrence risk, the risk that they may have another child with ASD. According to one participant,

*This [CMA testing] also would benefit us though because it would give us, are we going to have a 90% chance that we will have another child with autism? That would be something that would be interesting to know. (Male, White, Income: $35–$55K)*

*Confirming ASD Diagnosis*. As indicated in Table 2, three participants (6.7%) specified that they would utilize genetic testing to confirm their child's diagnosis of ASD.

#### 3.2.3. Barriers to Participating in CMA Testing

In addition to identifying participants' motivations to participate in CMA genetic testing, the research team also synthesized the barriers and reservations parents had before testing themselves and their children (Table 3).

*Cost of Testing*. The majority of participants (*n* = 33, 73.3%) were concerned with the cost of CMA testing. For example, one interviewee stated that

*This is a common barrier a lot of times insurance does not pay for genetic testing, and we do not have Medicaid, we have private insurance… Genetic testing can be very expensive, and most families do not have access to that kind of money. (Female, White, Income: $55–75K)*

*Risk/Pain Associated with CMA Testing*. Over one-third (*n* = 17, 37.8%) of participants mentioned that either the risk or pain associated with CMA testing would be a barrier of participating in genetic testing. One parent expressed his concern:

*My only concern would be he wouldn't want to take the blood out. You know he doesn't like doctors. He doesn't like needles, so that would be a challenge for him, to put him through that emotional rollercoaster…. (Male, White, $75–95K)*

*Fear of Results*. Fourteen participants (*n* = 14, 31.1%) mentioned that their fear of the results from the test would hinder them from participating in CMA testing. One of the participants has taken her son to undergo genetic testing for autism prior to our interview. She described her experience:

*It took months for the test results to get back. It took a while to return and it was nerve racking. (Female, White, $55–75K)*

A portion of participants (15.6%, *n* = 7) expressed that they had* little to no motivation* to test themselves or their child. Moreover, six participants (13.3%) stated that* transportation and/or scheduling issues* would be a barrier for them to participate in genetic testing. In addition, five participants (11.1%) articulated that their* lack of knowledge and understanding about CMA genetic testing* would prohibit them from participating. The illustrative quotes are shown in Table 3.

### 3.3. Discussion

This exploratory study highlights important findings and applications associated with parents' before-testing perceptions of CMA. The unique value of this empirical research is fourfold: first, our study was among the first community-based studies that investigated the willingness/motivations related to CMA genetic testing among parents with at least once child diagnosed with ASD. Previous investigations on genetic testing for autism were primarily conducted in clinical settings [12, 13, 16], where participants probably were prone to genetic research and had better access to care. Our community-based approach allows us to elicit broad opinions from diverse members within autism communities, particularly in rural areas.

Second, half of our participants were spouses who participated in our study and were interviewed individually. Past studies had investigated couples' perceptions of undergoing genetic testing for other conditions, such as bipolar disorders and cystic fibrosis, and found that couples had different viewpoints on having themselves or spouses tested for the genetic conditions run in the families [27, 28]. Our study found that spouses might blame each other related to who has passed on an autistic condition to their affected children. Some of them might feel guilty or blame themselves for being the culprit for their children's autism. Our study suggests that future education interventions should have a component in helping parents to alleviate negative emotions related to the test, such as guilt and blame, and focus more on locating the therapeutic and educational approaches using the information obtained from the test.

Third, our study was one of the first studies that addressed the perceptions particularly from low-income parents with diverse ethnicities; for instance, over one-third of our participants were Hispanic/Latino and all were Medicaid eligible. The average age of these Hispanic participants was below 40 (they are still at the reproductive age) and they showed keen interest in undergoing the test if it is available. Before our interviews, we asked parents which language they would prefer, most of them (13 out of the 14 Latino parents) preferred to be interviewed in Spanish. All of these participants mentioned that the cost of testing was the biggest barrier that might hinder their actual behavior of taking their children to undergo the test. Future educational interventions need to be linguistically sensitive for ethnic minorities. This design can enhance both their willingness to participate in the genetic research projects and also the comfort level of sharing information with the researchers.

Fourth, another salient finding of our study was that the overwhelming majority of our participants (almost 85%) showed interest and indicated motivation to either take their children to undergo CMA genetic testing or participate in the testing themselves. We found that parents' favorable positon towards CMA was slightly higher than two prior published studies that explored parents' attitudes and interest towards genetic testing for autism [21, 22]. Specifically, parents' favorable attitudes in CMA in our study were potentially explained by parents' perceived benefits from the usage of the test, such as finding a potential cause of ASD, getting prepared for having a child with ASD, learning more guidance on family planning, and early intervention. In contrast, in another study assessing parents' intention related to undergoing genetic testing for autism, parents mentioned that one of the most deciding factors for testing was the intent to reduce the anxiety level [21]. Given that parents were not aware of the availability of CMA for their affected children and families before participating in our study, therefore, the willingness to test might be imposed by insufficient understanding about these yet-unknown tests.

Parents' lack of awareness of CMA testing might reflect parents' limited access to medical professionals, such as genetic counselors, medical geneticists, and pediatricians who can order the CMA testing for the children affected with ASD. Also, genetic testing/assessments were usually not provided before children complete/go through a long diagnostic process. Parents might be referred by primary care practitioners to speak to a medical geneticist or genetic counselor after the initial diagnosis; however, none of the parents in our study, as we discussed, had been referred to any genetic specialists about CMA testing. This might be contributed to the limited resources due to the fact that parents in our study live in relatively underserved areas. For instance, in the specific geographic location where this study was conducted, no genetic counselors are available who might provide the information regarding undergoing CMA for parents and children with ASD. During our research, participating parents queried who they could ask about CMA and who could provide any counseling or education sessions for them. Given the limited number of genetic counselors, health education specialists are in a better position of providing educational resources to parents pertaining to CMA testing. Providers need to be aware of parents' lack of understanding of CMA, particularly among low-income parents. Our study also suggests the necessity of providing pretest counseling strategies that can be incorporated into the educational materials.

Several limitations of this research deserve attention when interpreting the results. First, our recruitment strategy aimed to reach parents of children with at least one child diagnosed with ASD. However, although we recruited participants from various ASD communities in ENC, the diagnosis and the severity level were self-reported by the participants themselves. We were not able to confirm the information claimed by parents. Second, parents' attitudes toward CMA genetic testing involved a hypothetical scenario of undergoing the test. We realize that our findings might differ from the actual situations where parents decide to test themselves or take their children to undergo CMA.

Genetic education and counseling tailored to parents of children with ASD are needed. As one of the first studies that explored parents' attitudes toward CMA, our results indicate a clear need for supporting the genetic services/education interventions for people and families affected with ASD, particularly in economically disadvantaged regions. Future studies might consider using a quantitative method with a larger sample size, such as surveys in various forms, to better understand parents' attitudes toward CMA genetic testing. In order to maximize the quality of genetic services related to CMA genetic testing, educational efforts should be implemented among clinicians, genetic counselors, and other health professionals, particularly in the context of precounselling stage prior to offering CMA to parents and their children with ASD.

## Figures and Tables

**Table 1:** Sample characteristics (*N* = 45).

Characteristics	*n* (%)
Participants' age: mean (range)	39.1 years (24–60)
Spouses' age: mean (range)	38.3 years (24–60)
Gender	
Females (mothers)	27 (60)
Males (fathers)	18 (40)
Number of children with ASD	
Boys	35 (79.5)
Girls	9 (20.5)
Race/ethnicity	
White	25 (55.5)
Hispanic/Latino	14 (31.1)
Black	4 (8.9)
American Indian/Alaskan native	1 (2.2)
Others	1 (2.2)
Education	
High school diploma or less	16 (35.6)
Some college	4 (8.9)
College graduate or above	25 (55.5)
Employment status	
Employed	32 (71.1)
Homemaker	11 (24.4)
Not employed	2 (4.4)
Annual household income	
<$15,000	7 (15.6)
$15,000–35,000	11 (24.4)
$35,001–$55,000	8 (17.8)
$55,001–$75,000	5 (11.1)
$75,001–$95,000	6 (13.3)
Over $95,001	8 (17.8)
Participants not born in USA	15 (33.3)
Child's age of diagnosis: mean (range)	4.3 years (1.5–13)
Severity level	
Level 1 (mild)	4 (9.1)
Level 2 (moderate)	23 (52.3)
Level 3 (severe)	11 (25)
Answer not provided	6 (13.6)

**Table 2:** Motivators for participants with at least one child with ASD to participate in CMA genetic testing (*N* = 45).

Theme	*n* (%)	Illustrative quote
Helping identify a potential cause of autism	21 (46.7)	*To know exactly where the origin of the problem of autism is that he has. (Female, Hispanic/Latino, Income:* <$15*K*) *The motivation would be to help the knowledge base better understand what causes autism… Having research like this can help the stigma of autism. (Male, American Indian/Alaskan native, Income:* >$95K)

Preparing for having another child with ASD	21 (46.7)	*I would like to know. I think it would be nice to know while you're pregnant with the child if the child is going to have autism… it would be a preparation tool. You know you would be able to prepare yourself, and to start preparing you and your family for what you're going to have to do when the child is growing up and prepare you for all the therapies and the cost of autism and the stress – that would be really helpful to know that information. (Male, White, Income: *$15–35*K*) *For me, if I wanted to have another child, I would be prepared if it was coming with autism…. (Female, Hispanic/Latino, Income: *$*15–35K)*

Family planning	18 (40)	*If I had done the test before I got pregnant, it would have been to make the decision if I would get pregnant or not or if I would have had this baby. (Female, Hispanic/Latino, Income: *<$*15K)*

Early intervention	17 (37.8)	*I can get help ahead of time… Instead of waiting until it is too late… so that he has to catch up. (Male, White, *$*75–95K)*

Research to benefit other families	15 (33.3)	*I think probably at this point in their [children with autism] lives the only reason why I would want to know is to help other people… but I think it would be especially at this point the testing would be more to help other people. (Female, White, Income:* >$*95K)*

Benefit their own child with autism	14 (31.1)	*I don't know that it will happen in my lifetime, but to help with drugs, chemicals, and could help even my son [with autism]. (Male, American Indian/Alaskan Native, Income: *>$*95K)*

Getting to know the recurrence risk	8 (17.7)	*I would personally do something like that, if I were to have another child I would want to know the risks, not that it would make a difference but it would be good to know. (Female, White, Income: *$*35–55K)*

Confirming autism diagnosis	3 (6.7)	*I would do it, [participate in CMA testing] not because I want to have more children, but to do away with doubts [that he has autism] and more than anything. (Male, Latino/Hispanic, Income: *$*35–55K)*

**Table 3:** Barriers for participants with at least one child with ASD that would keep them from participating in CMA genetic testing (*N* = 45).

Theme	*n* (%)	Illustrative quote
Cost	33 (73.3)	*The cost for a child with autism is already so expensive just for the therapy and getting help for the child. You know, adding another cost would be difficult. (Male, White, Income: *$*75–95K)* *Cost would be something to consider. I know a lot of genetic testing can be expensive. Umm cost would be a factor. (Male, White, Income: *$*35–55K)* *Maybe the expense of it. If it wasn't covered by insurance that might be something that would sway me. (Female, White, Income: *$*75–95K)*

Risk and pain associated with testing	17 (37.8)	*The concerns would be… how invasive it is and then the potential psychological issues it may cause them. (Male, White, Income: *$*35–55K)* *Maybe the concern would be about him getting his blood taken. It would be difficult for him. He doesn't like it when they take blood samples. He's afraid of needles. It would be difficult. (Female, Hispanic/Latino, Income: *$*15–35K)*

Fear of result	14 (33.3)	*I think it probably would open up a big can of worms as far as pointing fingers at where those genes came from. (Female, White, Income: >*$*95K)* *My concerns would be it [test result] comes back positive. (Female, Hispanic/Latino, *$*35–55K)*

Lack of motivation for testing	7 (15.6)	*Me personally, I wouldn't want to do it [undergo CMA testing]. I don't think it would change anything. It sort of why we resisted the diagnosis from the very beginning… I don't want a preconceived notion of who they're going to be. (Female, Hispanic/Latino, Income: *$*33–55K)*

Transportation and/or scheduling Issues	6 (13.3)	*We really don't have a whole lot of time to be able to do things like this testing. I do not want them out of school; they need that kind of structure. (Female, White, Income: *$*15–35K)* *The procedure and the timing because of my job I can't get off, because there are only two people in the office, me and my boss.… (Female, White, Income: <*$*15K)*

Lack of knowledge about testing	5 (11.1)	*I would need more information for them [children with autism] if they are going to do a blood test. (Female, Hispanic/Latino, Income: *$*35–55K)* *It would all depend on what the test will give you. If the test says, ‘Your son might have autism, your son might not,' I wouldn't want that because I wouldn't want to go looking for the problem without a cause. If you can give it to me definitively, ‘yes your son has the markers. He's going to be expected by the autism spectrum in some way.' Then sure, I might want to know that up front. (Female, White, Income: >*$*95K)* *I don't know. I don't know enough about the test. (Male, White, Income: *$*75–95K)*
